# Supplementary material for: Syringin and Phillygenin—Natural Compounds with a Potential Role in Preventing Lipid Deposition in Macrophages in the Context of Human Atherosclerotic Plaque
Source: Int J Mol Sci. 2025 Jul 4;26(13):6444. doi: 10.3390/ijms26136444 (PMC12250599; doi:10.3390/ijms26136444)
Supplement: Supplementary file 1 [file ijms-26-06444-s001.zip › ijms-3630398-supplementary.pdf]

Supplementary Materials for

Syringin and phillygenin – natural compounds with a potential role in preventing lipid deposition in macrophages in the context of human atherosclerotic plaque.

Filipek A.[1]*, Sadowska A.[1], Skłodowska M.[1], Muskała M.[1], Czepielewska E.[2]

[1] Chair and Department of Pharmaceutical Biology, Medical University of Warsaw, Banacha 1, 02-097 Warsaw, Poland

[2] School of Health and Medical Sciences, Vizja University, Okopowa 59, 01-043 Warsaw, Poland

*Corresponding author:

Chair and Department of Pharmaceutical Biology, Medical University of Warsaw, Banacha 1, 02-097 Warsaw, Poland

Tel.: +48 225720983

E-mail address: agnieszka.filipek@wum.edu.pl

ORCID: https://orcid.org/0000-0002-1351-3687

Document S1

A. Cytotoxicity [%] of syringin or phillygenin (10 µg/mL, 20 µg/mL, 50 µg/mL) and cholesterol (20 µg/ml), as well as kaempherol (20 µg/mL) on macrophages. Data from 3 experiments ± SEM (*p>0.05*).

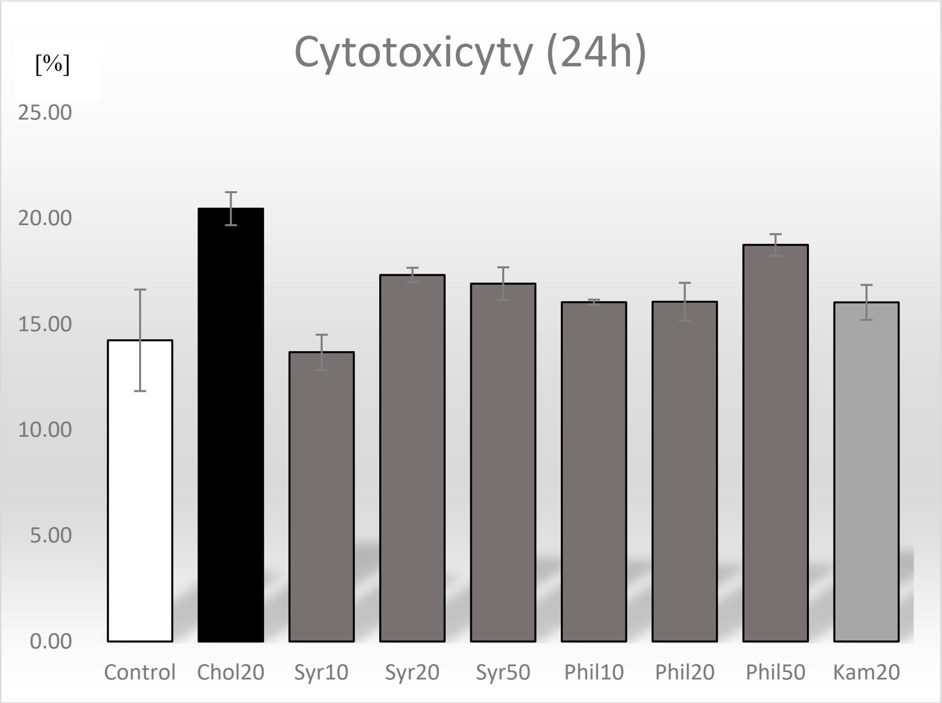

Control - macrophages incubated without compounds. Chol20 - macrophages incubated with cholesterol 20 µg/ml; Syr10, Syr20, Syr50 – macrophages incubated with cholesterol (20 µg/mL) for 24 h and then incubated with syringin (10 µg/mL, 20 µg/mL, 50 µg/mL) for the next 24 h; Phil10, Phil20, Phil50 – macrophages incubated with cholesterol (20 µg/mL) for 24 h and then incubated with phillygenin (10 µg/mL, 20 µg/mL, 50 µg/mL) for the next 24 h. Kam20 - macrophages incubated with cholesterol (20 µg/mL) for 24 h and then incubated with Kaempferol (20 µg/mL).

B. Cytotoxicity [%] of rosiglitazone (1 µg/mL) with syringin or phillygenin (10 µg/mL, 20 µg/mL, 50 µg/mL) and cholesterol (20 µg/ml), as well as kaempherol (20 µg/mL) on macrophages. Data from 3 experiments ± SEM (*p>0.05*)

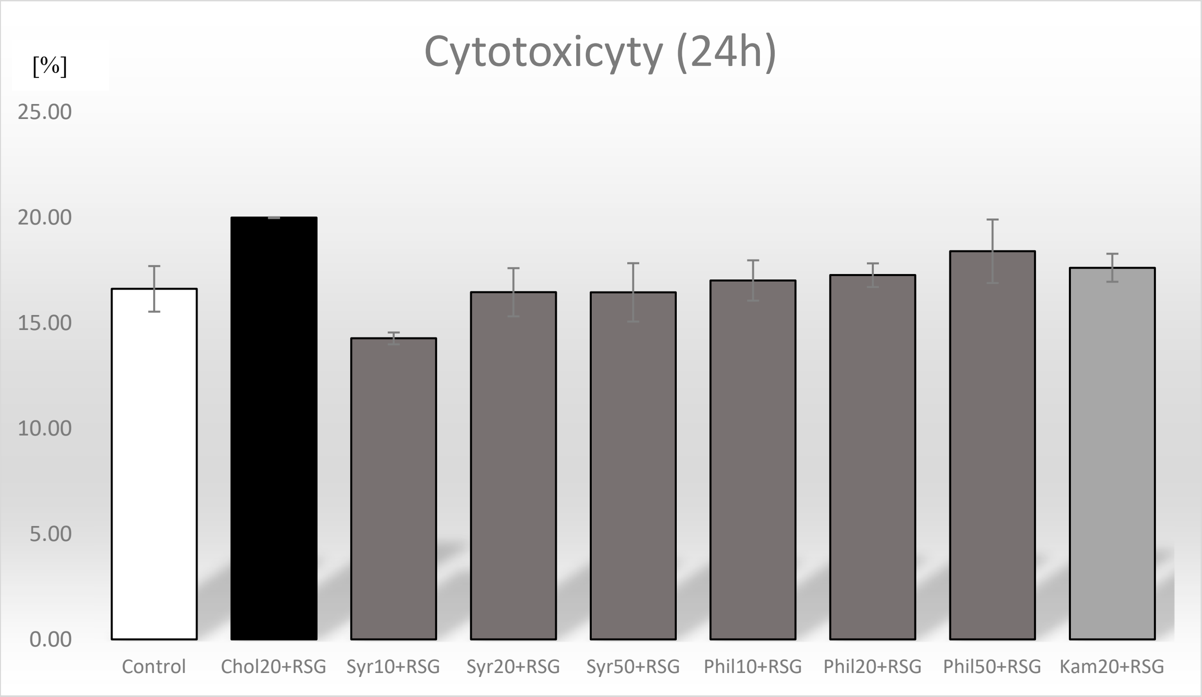

Control - macrophages incubated without compounds. Chol20+RSG - macrophages incubated with cholesterol 20 µg/ml and rosiglitazone (1 µg/mL); Syr10, Syr20, Syr50 – macrophages incubated with rosiglitazone (1 µg/mL) and cholesterol (20 µg/mL) for 24 h and then incubated with syringin (10 µg/mL, 20 µg/mL, 50 µg/mL) for the next 24 h; Phil10, Phil20, Phil50 – macrophages incubated with rosiglitazone (1 µg/mL) and cholesterol (20 µg/mL) for 24 h and then incubated with phillygenin (10 µg/mL, 20 µg/mL, 50 µg/mL) for the next 24 h. Kam20 - macrophages incubated with rosiglitazone (1 µg/mL) and cholesterol (20 µg/mL) for 24 h and then incubated with kaempferol (20 µg/mL).
